# Coupling Coordination Analysis of Urban Development and Ecological Environment in Urban Area of Guilin Based on Multi-Source Data

**DOI:** 10.3390/ijerph191912583

**Published:** 2022-10-02

**Authors:** Taolin Liu, Chao Ren, Shengguo Zhang, Anchao Yin, Weiting Yue

**Affiliations:** 1 College of Geomatics and Geoinformation, Guilin University of Technology, Guilin 541004, China; 2 Guangxi Key Laboratory of Spatial Information and Geomatics, Guilin University of Technology, Guilin 541004, China

**Keywords:** urban area of Guilin, night-time light index, remote sensing ecological index, coupling coordination degree model, urbanization and ecological environment

## Abstract

Urban development in developing regions increases ecological and environmental pressures. Few annual ecological studies have been conducted on tourist-oriented cities. Guilin is famous as an international tourist destination in Chine. Analyzing its coupling coordination between urbanization and ecology is vital for subsequent sustainable development. This paper constructed a night-time light index (NTLI) based on DMSP/OLS, NPP/VIIRS night-time light data in response to these problems. The remote sensing ecological index (RSEI) model was established in this study by using four indexes: greenness, wetness, dryness and heat. The coupling coordination degree model (CCDM) was built. From the dynamic time-series changes of CCDM, the urban development and ecological environment of the urban area of Guilin, from 2000 to 2020, were analyzed. The results showed that the urban area of Guilin’s urbanization had developed rapidly over the past 20 years. NTLI in 2020 was 7.72 times higher than in 2000. The overall ecological quality of the main urban area of Guilin has improved significantly, while local ecological pressure in Lingui District has increased. CCDM has shifted from low to high coupling coordination, and the relationship between urban development and the ecological environment has improved. The method of annual spatial-temporal analysis of urban ecology in this paper can be applied in similar studies on other cities, and the results obtained for Guilin have reference value for future urban planning and environmental protection work.

## 1. Introduction

China’s urban economic development has been miraculous since the reform and opening up, over 40 years ago. This reform and opening up is the policy of domestic reform and opening up that China began to implement at the Third Plenary Session of the Eleventh Central Committee in December 1978. Between 1980 and 2020, China’s urbanization rate increased from 19.4% to 63.9% (http://www.stats.gov.cn/ (accessed on 6 August 2021)). Rapid socio-economic development has greatly enhanced the living standards and the life quality of the Chinese people. However, in the early stage of the reform, the focus on single economic development ignored ecological protection, which is also a problem that most developing countries will encounter in social development [1,2]. China’s rapid urban economic development has caused a serious ecological impact [3]. The increase in urban population, urban area expansion and a decrease in green vegetation have increased ecological pressure [4,5]. The Chinese government has implemented ecological civilization and sustainable development strategies in order to solve the environmental problems existing in economic development [6]. Ecological changes vary from one environmental region to another, so it is necessary to know the relationship between urban area and ecology for different typical regions.

Modern urbanization consists of population urbanization, industrial structure, geographical space and social civilization [7]. In social development, urban development and ecological changes are dynamic, mutually interactive and coupled relationships [8,9]. Human activities led to a reduction in natural resources and an increase in urban land use, reducing the green area. At the same time, the natural ecosystem gradually transformed into an urban ecosystem [10]. The CCDM of urban development and ecology is different in different contexts [11]. At present, studies on the CCDM of urban ecology focused on large-scale regions and long time series, but there are few studies on the annual CCDM of cities in typical tourism regions. Under vigorously developing ecotourism in China, the continuous impact of human activities and economic development on the ecological environment of tourist cities is more worthy of our attention. Previous studies on urban development with land changes, through the transformation of other land types into urban built-up areas, can reflect the level of urbanization [12,13]. Building indices can monitor issues related to urban sprawl and environmental changes [14]. However, cities have shifted to multiple development elements with urban development, and a single indicator is no longer a good representation of urban development. The elements of urban development include various dimensions, such as population quality, quality of life, economic quality, environmental quality, and urban quality [15]. It is crucial to find a comprehensive indicator that effectively represents the city’s development in this context. NTLI is closely related to population, GDP, development density, development pattern, degree of social development, and functions of cities/developed areas levels, which is a comprehensive indicator that can reflect urbanization and better represent urban development [16,17,18,19]. Currently, night lighting data is widely used for urban population, economic development, urban pollution, and electricity consumption assessment.

With the continuous application of remote sensing technology, remote sensing images were widely used in ecological monitoring and evaluation [20]. Compared to traditional methods of ecological evaluation through data almanacs, remote sensing technology has enormous advantages in terms of ecological visualization and spatial distribution. Several evaluation indicators have been proposed for the evaluation of ecological environments. Ecosystem health was evaluated through vegetation, such as vegetation coverage, normalized difference vegetation index (NDVI), and biomass [21,22,23]. Exploring the impact of CO_2_ release on global temperature rose by studying population increase [24]. The inversion of water quality parameters allowed for the evaluation of water ecosystems and effective monitoring of water resources [25]. Crop and industrial distribution indicators can reflect artificial ecosystem health [26]. These indicators can achieve good results in eco-regional studies, but it is not easy to evaluate complex ecological environments with a single evaluation indicator, due to the extreme complexity of ecosystems. RSEI is a comprehensive ecological evaluation index of multiple indicators [27]. RSEI can objectively and rapidly evaluate the ecological quality of the study area [28]. According to research, NTLI can better represent the level of urban development, and RSEI can represent the ecological environment condition. Therefore, by introducing CCDM, the analysis of the two together can reflect the relationship between urban development and ecological changes. However, previous scholars had conducted a single study using only historical urban data or remote sensing data. Few studies combined historical urban data with remote sensing data for analysis. Using urbanization data and agricultural ecological environment historical data to build a coupling and coordination model, the paper empirically studied the spatio-temporal differentiation of the coupling and coordination development of China’s new urbanization and agricultural ecological environment [29]. The paper used multi-source remote sensing data to build ecological environment indicators and urbanization development indicators, and studied the coupling and coordination relationship between cities and ecological environment in eastern China [30]. In the analysis of urban ecological coupling coordination, single historical data or remote sensing data will cause incomplete analysis. For example, more population, GDP, social welfare, or other social data, should be considered in the analysis of urban change [31]. Therefore, we combined historical and remote sensing data for an annual coupled and coordinated analysis of the relationship between urban development and ecological changes.

Guilin’s ecological changes are significant as a famous international tourist city. The urban area of Guilin was the economic, cultural, and main tourist center of Guilin. After 20 years of development and construction, the urban structure has changed. Therefore, this paper constructed NTLI based on the night-time light data of the urban area of Guilin. Combined with the multispectral remote sensing images, the greenness index, wetness index, dryness index, and heat index, were built. We used principal component analysis (PCA) to construct RSEI. NTLI and RSEI were used as a CCDM subsystem system. The study and analysis of the annual CCDM of urban development and ecological environment in the main urban area for 20 years, from 2000 to 2020, to provide data reference for the next phase of ecological landscape pattern planning in Guilin’s main urban area.

## 2. Study Area and Materials

### 2.1. Study Area

**Figure 1:**
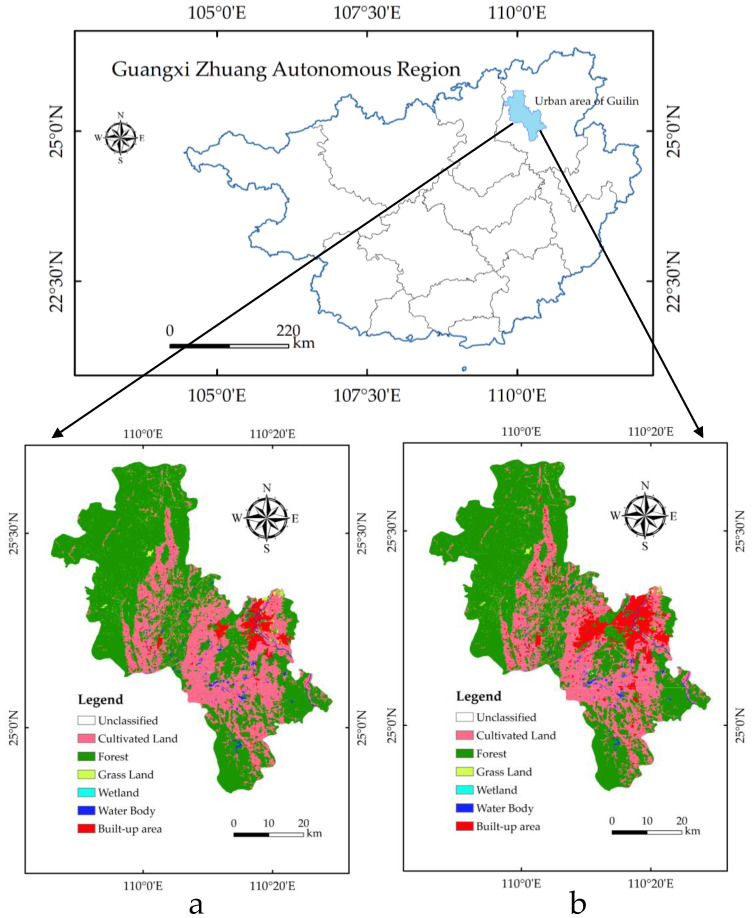
Land type map of the study area. (**a**) is the land type map of the study area in 2010. (**b**) is the land type map of the study area in 2020. Data source: National Geographic Information Resource Directory Service System (https://www.webmap.cn/main.do?method=index (accessed on 15 September 2022)).

The central city of Guilin is located in the northeastern part of the Guangxi Zhuang Autonomous Region (Figure 1). It is a typical karst-type landscape with high vegetation coverage. The urban area of Guilin contains six districts: Xiangshan District, Qixing District, Xiufeng District, Lingui District, Diecai District, and Yanshan District, with a total area of 2854 km^2^ and a resident population of over 1.5 million. The annual average temperature of the central city is 19.0 °C, and the annual average precipitation is 1895 mm. In 2019, the GDP of Guilin was CNY 210.556 billion, an increase of 6.5% over the previous year at comparable prices. Among them, the added value of the primary industry increased by 6.0%. The added value of the secondary industry increased by 7.2%. The added value of the tertiary industry increased by 6.5%. By the end of 2017, Guilin had established 12 nature reserves, with a total area of 427,000 hectares, accounting for 15.36% of the city’s land area, including four national nature reserves and seven autonomous region level nature reserves.

### 2.2. Data Resources and Processing

Datasets in the paper consist mainly of night lighting data and Landsat series remote sensing images. The data was downloaded from the Google Earth Engine (GEE) cloud platform and the time series was 2000–2020. GEE is a cloud computing platform for storing satellite data and batch processing satellite data. GEE stores over 40 years of remote sensing data worldwide, including the Landsat series, Sentinel, MODIS, and other data. GEE platform offers Python and JavaScript client libraries, allowing users to freely write code in the GEE code editor and then transfer it to the cloud for big data-parallel computing. The platform improved the efficiency of data processing. GEE’s high storage and cloud computing capabilities enable rapid processing and the saving of long-duration remote sensing data for download [32].

#### 2.2.1. Night-Time Lighting Data

**Figure 2:**
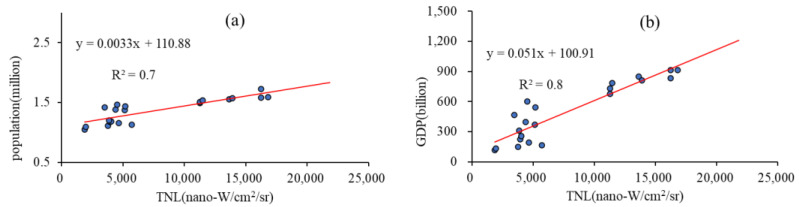
Validation of TNL (total night-time light) with population and GDP correlations. (**a**) TNL-population correlation. (**b**) TNL-GDP correlation.

DMSP/OLS data was taken out of service in 2013 and replaced by NPP/VIIRS data. Therefore, the DMSP/OLS (https://www.ngdc.noaa.gov/eog/dmsp/downloadV4composites.html (accessed on 16 October 2021)) annual average night-time light data from 2000 to 2013 and the NPP/VIIRS (https://www.ngdc.noaa.gov/eog/viirs/download_DNb_composites.html (accessed on 17 October 2021)) from 2013–2020 were downloaded for the study. NPP/VIIRS data have higher spatial resolution and a more comprehensive spectral range, with enhanced low-light detection and the ability to identify more acceptable lights. Two types of night lighting data also differ in how they respond to light. DMSP/OLS data reflects light intensity by recording brightness values, while the NPP/VIIRS data relies on night radiation intensity. Two data need to be corrected for each other to construct NTLI [33]. Firstly, NPP/VIIRS data resampling to a spatial resolution of 1 km. A conversion model was constructed using the 2013 DMSP/OLS and NPP/VIIRS public monthly data, and the conversion model was used to convert the NPP/VIIRS to obtain DMSP/OLS. Historical population and economic data from the urban area of Guilin were collected to analyze the reliability of the corrected night-time lighting. The validation of the effect through Figure 2 showed that the R^2^ of population and GDP with the total night-time light (TNL) values is 0.72 and 0.83, respectively. The high correlation described that DMSP/OLS and NPP/VIIRS were fine corrected for each other. It gave a uniform standard of night lighting data that could be used to construct NTLI.

#### 2.2.2. Optical Remote Sensing Images

Optical remote sensing images data included the Landsat TM, Landsat ETM, and Landsat OLI series. Considering that the weather in Guilin was highly variable and cloudy, which had a bad impact on the experiment, stable autumn images were selected as the data for this study. We reduced the impact of cloud cover on image quality at the GEE through cloud screening and the cloud removal function. The raw images were pre-processed prior to the calculation of the four indicators. The images were first radiometrically calibrated and atmospherically corrected to remove the effects of atmospheric and lighting factors on feature reflections. Finally, the images were cropped using the vector boundary of the urban area of Guilin to obtain an image map of the study area.

RSEI is a composite indicator based on four indicators: greenness, wetness, dryness, and heat, which evaluate the ecological situation. The experiment was based on normalized difference vegetation index (NDVI), tasseled cap wetness (TCW), normalized difference built-up and soil index (NDBSI) and land surface temperature (LST) to construct RSEI. In order to have a uniform outline quantity for each index, each indicator needs to be normalized separately, as follows:

(1) Greenness indicators

NDVI reflects the vegetation cover and health of the study area [34]. It is calculated using the expression Equation (1):

(1) $$NDVI=\frac{Nir-Red}{Nir+Red}$$

Among them, Red and Nir are the red and near-infrared reflectance, respectively.

(2) Wetness indicators

Tassel cap change is widely used in soil water content, land cover change, etc. [35], and is calculated as Equation (2):

(2) TCW=a Blue+b Green+c Red+d Nir+e Swir1+f Swir2

Among them, Blue, Blue, Red, Nir, Swir1 and Swir2 stand for blue-band, green-band, red-band, near-infrared, the first wave of the short-wave infrared and the second wave of the short-wave, respectively. *a*, *b*, *c*, *d*, *e,* and *f,* ask for the reflectance coefficients of the corresponding bands, which vary for different sensor values [36,37,38]. Table 1 lists the tassel cap transformation coefficients for different sensors.

(3) Dryness index

The dryness index, consisting of index-based built-up index (IBI) and soil index (SI), is an important indicator of the degree of drying of impervious surfaces [39,40], as expressed in Equations (3)–(5):

(3) $$IBI=\frac{\frac{2Swir1}{Swir1+Nir}-\left(\frac{Nir}{Nir+Red}+\frac{Green}{Green+Swir1}\right)}{\frac{2Swir1}{Swir1+Nir}+\left(\frac{Nir}{Nir+Red}+\frac{Green}{Green+Swir1}\right)}$$

(4) $$SI=\frac{\left[\left(Swir1+Red\right)-\left(Nir+Blue\right)\right]}{\left[\left(Swir1+Red\right)+\left(Nir+Blue\right)\right]}$$

(5) $$NDBSI=\frac{IBI+SI}{2}$$

where Blue, Green, Red, Nir, Swir1 and Swir2 represent the blue, green, red, near infrared, the first wave of the short-wave infrared and the second wave of the short-wave infrared, respectively.

(4) Heat index

The heat indicators, which reflects land surface temperature changes [41], was calculated using the atmospheric correction method using the formula. LST is given in Formulas (6) and (7):

(6) $$B\left(T_{s}\right)=\frac{L_{\tau }-L_{↑}-\beta \left(1-\theta \right)L_{↓}}{\beta \theta }$$

(7) $$LST=\frac{K_{2}}{\ln\frac{K_{1}}{B\left(T_{s}\right)+1}}$$

Among them, $L_{\tau }$ is Landsat thermal infrared band, $L_{↑}$ and $L_{↓}$ represent the upward and downward atmospheric radiance, respectively, *β* is the transmittance of the thermal infrared band, *θ* is the surface specific emissivity, $K_{1}$ and $K_{2}$ are the calibration coefficients, respectively, and LST is the surface temperature.

## 3. Models and Methods

**Figure 3:**
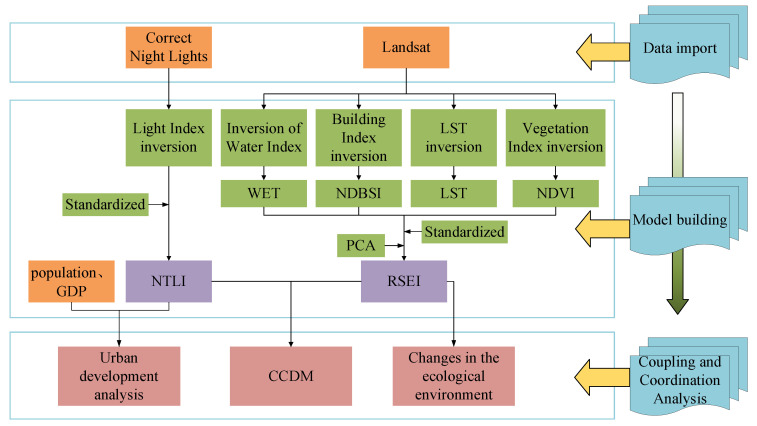
The technology roadmap for this study.

In this paper, we first pre-processed the light data and remote sensing images data and then inverted each index to obtain NTLI and RSEI. CCDM was introduced to analyze the coupling coordination of NTLI and RSEI, and trended analysis of remotely sensed ecological indices (Figure 3).

### 3.1. NTLI

The average night-time light intensity is a composite representation of the level of urbanization, so it is appropriate to use the average night-time light intensity to analyze and evaluate urbanization [42]. NTLI was constructed based on the regional average light attribute and regional light area attribute, and there was a significant correlation between the light index and the composite indicator of urbanization level [43]. We combined new lighting data obtained from the DMSP/OLS and NPP/VIIRS inter-calibration simulations in this paper and the actual situation of urbanization development in the urban area of Guilin. This paper used the regional average light intensity and regional light area to construct NTLI to reflect the level of urbanization development in the study area. The calculation formulae are as follows Equation (8):

(8) $$NTLI_{i}=I_{i}\times W_{1}+S_{i}\times W_{2}$$

Among them, $NTLI_{i}$ is NTLI of study area i, $I_{i}$ is the average night-time light intensity, $S_{i}$ is the night-time light area covariate, $W_{1}$ and $W_{2}$ correspond to the weights, combined with the thesis and the actual situation in the study area of this paper for careful consideration, $W_{1}$ and $W_{2}$ in the paper are 0.8 and 0.2, respectively. Where the average light intensity at night is calculated by Equation (9):

(9) $$I_{i}=\overset{63}{\underset{j=1}{\sum }}DN_{j}\times \frac{m_{j}}{N\times 63}$$

where $DN_{j}$ is the night light gray value, $m_{j}$ is the total number of image elements for the drinking gray value, and N is the total number of image elements in the study area.

Area lighting area can be calculated using Equation (10):

(10) $$S_{i}=\frac{S_{n}}{S_{k}}$$

where $S_{i}$ is the total area of light elements in the study area and $S_{k}$ is the total area of the study area.

### 3.2. RSEI

RSEI is a comprehensive index proposed to evaluate the ecological conditions based on four indicators: greenness, wetness, dryness, and heat. These are closely related to vegetation cover, vegetation and soil moisture, building, bare soil distribution, and surface temperature, respectively. The four indicators are four ecologically important factors of ecological change. The article used principal component analysis to construct RSEI based on the four indices: NDVI, TCW, NDBSI, LST. In order to make the four indices have a unified dimension, it is necessary to normalize the four indices so that their values are in [0, 1]. These are shown in Equations (11)–(13).

(11) $$N_{i}=\frac{I-I_{min}}{I_{max}-I_{min}}$$

(12) $$RSEI_{0}=\left\{PC1\left[f\left(NDVI,Wet,LST,NDBSI\right)\right]\right\}$$

(13) $$RSEI=\frac{RSEI_{0}-RSEI_{0\_min}}{RSEI_{0\_max}-RSEI_{0\_min}}$$

where: $N_{i}$ is the normalized value of the four indices, I is the image element value of the corresponding index, $I_{min}$ is the minimum image element value of the index, $I_{max}$ is the maximum image element value of the index. A higher *RSEI* value means a better ecological environment for the area, a lower value means a worse ecological environment.

### 3.3. CCDM

Coupling (C) can reflect the degree of the close connection between system factors and effectively predict the development pattern and evolution of the system. *C* is calculated as Equation (14). However, *C* as a whole hardly reflects the actual connection between ecological change and urban development and has a dynamic and unbalanced character. *CCDM* can objectively and realistically evaluate the coupling relationship between regional ecology and urban development in the urban area of Guilin [44]. *CCDM* is calculated as shown in Equations (15) and (16):

(14) $$C={\left[\frac{u_{1}\times u_{2}}{{\left(u_{1}+u_{2}\right)}^{2}}\right]}^{\frac{1}{2}}$$

(15) $$T=a\times u_{1}+b\times u_{2}$$

(16) $$CCDM={\left(C\times T\right)}^{\frac{1}{2}}$$

where T is the integrated urbanization and ecology reconciliation index. $u_{1}$ and $u_{2}$ represent NTLI and RSEI. A and b represent the weight of urban development and ecology in the system, in this study the level of urbanization and ecology are equally important, a = b = 0.5.

As shown in Table 2, we divide CCDM into three categories and nine subcategories, according to the actual situation in the main urban area of Guilin.

## 4. Results and Analysis

### 4.1. Analysis of Changes in Urban Sprawl

**Figure 4:**
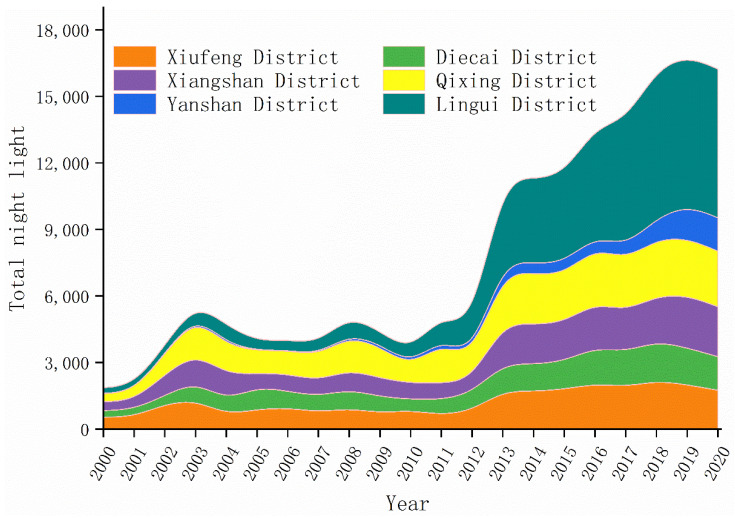
Changes in night-time light intensity in the urban area of Guilin from 2000 to 2020.

The analysis of night-time lighting changes provided a visual insight into the changes in urban expansion over the last 20 years in the main city of Guilin. In order to reflect the development of lighting in various regions, we calculated the total night light in each region of Guilin main urban area through each pixel value of night lighting images in ArcGIS. The results are shown in Figure 4. Figure 4 showed that the intensity of night-time lighting has been increasing from 2000 to 2020. The total amount of night-time lighting in 2020 has increased by 772% compared to 2000. The urbanization of Guilin’s main urban area has been remarkable. In 2002, the total amount of night lights in the main urban area of Guilin increased significantly. Because the Guilin government strengthened construction around the urban area, industrial production continued to grow steadily and rapidly. In 2012, Guilin received 32.93 million domestic and foreign tourists, an increase of 18.1%, which is an important reason for the increase of the total amount of night lights in Guilin in 2012. As Guilin is a tourist city, its industrial level is low. Under the condition of vigorously developing tourism, Guilin has basically built up an international tourist attraction and realized the upgrading and development of quality. This is closely related to the geographical environment of Guilin. The urban level in Xiufeng, Diecai, Xiangshan, and Qixing districts, has steadily improved. Yanshan District and Lingui District developed slowly until 2012 and then developed at a high rate with a marked increase in light intensity. From the perspective of the urbanization rate of each district and county, Xiufeng District has the highest urbanization rate of 100%, and Lingui District has the lowest urbanization rate of 57.43%. Because Lingui District and Yanshan District belong to the new construction area, their urbanization level and urbanization area are relatively low, which are also the key development objects of Guilin in the future.

In order to better understand the relationship between changes in night-time light intensity and urban social development, IPD, GDP per capita, and urbanization rate were calculated in this paper (Table 3). Combined with the data analysis in Table 3, the development of urbanization in the urban area of Guilin was divided into two main periods. The first phase was a period of rapid development from 2000 to 2015. The average NTLI rose from 0.05 in 2000 to 0.75 in 2015, with an average annual growth rate of 5%. GDP maintained a high growth trend. The urban population has increased rapidly, especially since the introduction of China’s poverty eradication policy. The urban population increased from 2,312,900 in 2015 to 2,600,000 in 2019, an increase of 17.62%. In 2020 the average NTLI reached 0.90. GDP has remained stable and consistently rising over the last five years. There was good consistency between changes in night-time light density and changes in IPD and GDP urbanization.

We try to analyze Table 3 and Figure 4 together and discover an interesting finding. The change trends of night light density and NTLI are roughly the same, but different from the former two. The change trends of population density, urbanization rate and per capita GDP are the same, and these are in a state of continuous growth. We believe that it is also more in line with the state of urbanization. It is proved that more social data should be added at the same time instead of just using NTLI to represent the level of urbanization.

### 4.2. Ecological Change Analysis

In the paper, based on the calculation of four indicators, NDVI, TCW, NDBSI, and LST, RSEI was constructed using principal component analysis to analyze the ecological environment of the urban area of Guilin from 2000 to 2020. PCA allows for the effective integration of valid information from several indicators, and the contribution margin allows for the weighting of each indicator in the RSEI. The calculation of RSEI requires the use of principal components. Therefore, we use Landsat images to calculate NDVI, TCW, NDBSI, and LST, respectively. Then, the four indicators are combined into a four-dimensional matrix to calculate the covariance matrix, and then the eigenvalue and contribution rate of the covariance matrix are calculated. The results are shown in Table 4. From the results of the partial PCA in Table 4, the contribution of the eigenvalues of the first principal component (PC1) analysis for 2000, 2010, and 2020, were 97.16%, 95.15%, and 95.94%, respectively, which were all greater than 95%, indicating that PC1 integrates most of the information of the four covariates. The contribution of PC2–4 was low and the error was enormous, so PC1 was chosen to calculate RSEI in this paper.

In order to better understand the impact of these four indicators on RSEI, we calculated the average value and standard of each indicator using Table 4 eigenvalue and contribution rate. The statistical results are shown in Table 5. From the average values of the four covariates in Table 5, the indicators of greenness and wetness positively contributed to the ecological environment, while the indicators of dryness and heat had a negative impact. Because of the high vegetation cover in the urban area of Guilin and the city’s policy of strengthening afforestation and returning farmland to forest, the positive effects of greenness and wetness were generally higher than the harmful effects of dryness and heat. The greenness indicator increased from 0.457 in 2000 to 0.688 in 2020, an increase of 23.1%. It indicated that the increase in vegetation area had a positive impact on the ecological improvement of the urban area of Guilin.

**Figure 5:**
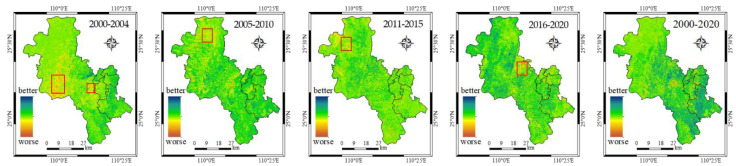
Change map of RSEI in the main urban area of Guilin from 2000 to 2020 with five-year intervals. The red box indicates the area with a large range of rsei reduction in the past five years, which refers to the area with poor ecological environment.

In order to visualize the ecological changes in Guilin urban area, the area of RSEI difference was calculated by the experiment using Figure 5. The range of RSEI difference was [–1, 1], and it was divided into nine categories within the range for hierarchical area statistics. According to the ecological situation in the main urban area of Guilin, the nine grades were: abnormal poor (−1~−0.35), significant poor (−0.35~−0.25), general poor (−0.25~−0.15), slightly poor (−0.15~−0.05), unchanged (−0.05~0.05), slightly good (0.05~0.15), generally good (0.15~0.25), significantly good (0.25~0.35), and abnormally good (0.35~1). The area and percentage age of ecological environment change in Guilin urban area in different periods are calculated, as shown in Table 6.

The statistical results in Table 6 showed that ecological deterioration and improvement were mainly reflected in a slight decrease and a slight increase, with no significant amount of ecological improvement or deterioration. Over 35% of the area RSEI remained unchanged (−0.05~0.05). The next largest changes were generally better (0.15~0.25) and generally worse (−0.25~−0.15), with a higher proportion of generally better than generally worse. Greater ecological change was concentrated in slightly better (0.05~0.15) and slightly worse (−0.1~−0.05). There were fewer areas of significant change, with the basic proportion of significantly better (0.25~0.35) and significantly worse (−0.35~−0.25), both being around 0.5%. There were even fewer abnormally good (0.35~1) changes and abnormally bad (−1~−0.35). It indicated that ecology needs long-term management and protection. In the 2016–2020 time period, the area of ecologically better areas in urban areas was 1279.5 km^2^, an increase of 718.9 km^2^ compared to 2000–2004. 2000–2020, the area of ecologically better areas in Guilin urban areas was 1617.91 km^2^, accounting for 55.36% of the total area and 6.79% of the deteriorated area, with overall improvement of ecological environment.

In order to show the ecological changes of the study area in the past 20 years in more detail, we draw an ecological change map every five years to reflect the improvement or deterioration of the ecology. Specifically, the RSEI of the last year minus the RSEI of the first year. If it is negative, it indicates that the ecological environment has deteriorated. If it is positive, it means that the ecology is better. In Figure 5 the results are obtained. Figure 5 spatially illustrated the distribution of ecological changes at five-year intervals. Guilin’s urban area has shown an overall ecological bias towards improvement over the past two decades but showed local spatial heterogeneity. The ecology of Lingui District showed a decreasing trend from 2000 to 2004, while some old urban areas, such as Qixing District and Xiangshan District, continued to improve, with a small increase in RSEI values.

**Figure 6:**
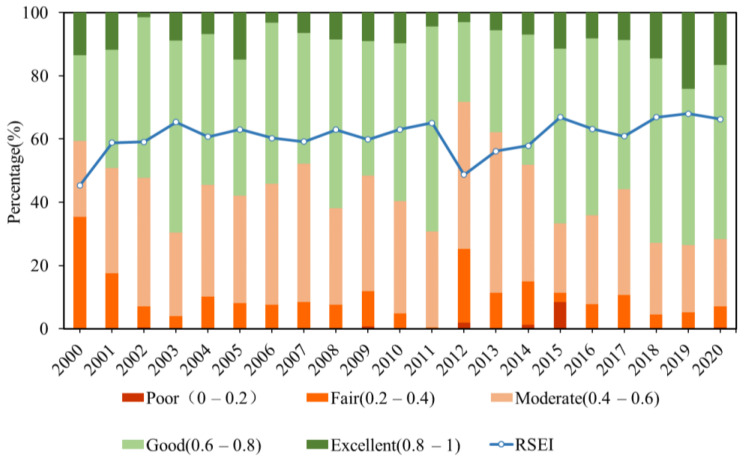
Percentage of RSEI classes and changed in RSEI between years.

It is worth noting that we want to know the proportion of each type of ecological environment quality. We can easily understand the changes of environmental quality at all levels in the main urban areas of Guilin in the past 20 years. RSEI was divided into five grades at intervals of 0.2, asking for poor (0~0.2), fair (0.2~0.4), moderate (0.4~0.6), good (0.6~0.8), and excellent (0.8~1), respectively. As shown in Figure 6, the proportion of medium and good grades is more significant and stable, the high-quality ecological environment is on the rise, and the poor are gradually decreasing. The reason for this was that the quality of the medium ecological environment has gradually turned into good and high quality, the poor ecological environment has gradually improved, and the overall RSEI increased.

The distribution of local ecological changes in Figure 5 showed that after 20 years of comprehensive management and ecological transformation, the RSEI values of some urban areas with poorer environments have increased. The ecological quality of the Yanshan district in the south of the main urban area has improved. However, with the development and construction of the northern part of the urban area of Guilin, and the expansion of the urban area, some agricultural land or non-construction land has been developed into urban construction land, and the quality of the ecological environment in some areas has deteriorated. Therefore, in the future, attention needs to be paid to ecological protection and strengthening of greening in the area.

### 4.3. Analysis of the Coupling and Coordination of Urban Development and Ecological Change

**Figure 7:**
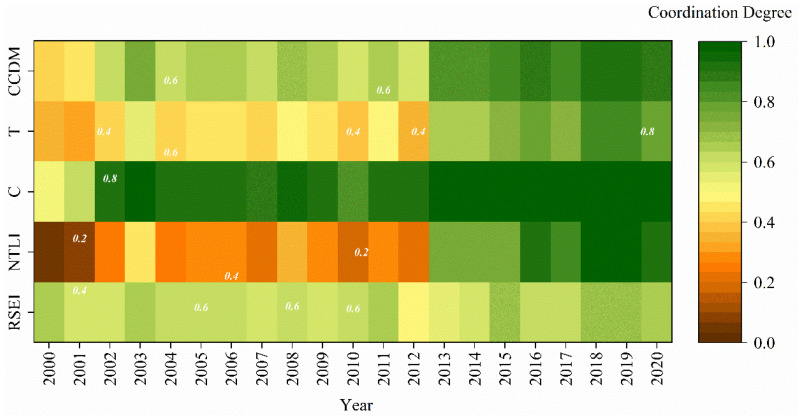
Interannual variation in urban ecological indicators.

By constructing C and CCDM, the C and CCDM of urbanization and ecological environment in the study area, from 2000 to 2020, were calculated. We have calculated CCDM using Formulas (14)–(16), to better understand the coupling and coordination between urban development and ecology. The experimental statistics of each index data of the main urban area of Guilin every year were carried out for comprehensive urban ecological analysis. Figure 7 showed the changes in the various indicators. The CCDM of the urban area of Guilin has increased over the past 20 years from 0.37 in 2000 to 0.78 in 2020. Cities and ecology were in a phase of rapid coupled development. In particular, the Corona Virus Disease (COVID-19) impact in 2020 has led to Guilin being in an economic downturn in tourism, catering, and consumerism. To a certain extent, it had affected the urbanization of Guilin’s main urban area. With control of COVID-19 in China, the number of tourist trips to Guilin will increase. The recovery of Guilin’s various economies will continue so that economic development will enter a more rapid phase of development in the next phase. Urbanization will be accelerated and will develop in conjunction with the ecological environment. C in 2020 was 0.47 higher than in 2000, showing a clear trend of first increasing and then remaining stable, reflecting the interaction between urban development and the ecological environment. C has always been higher than the CCDM, but the difference gradually decreases. It indicates that urban ecology and urban development had always been positively correlated, while CCDM tended to develop in line with the related economic construction and government policies. In terms of T, the urban area of Guilin is developing slowly, which is in line with the social development of the urban area of Guilin.

**Figure 8:**
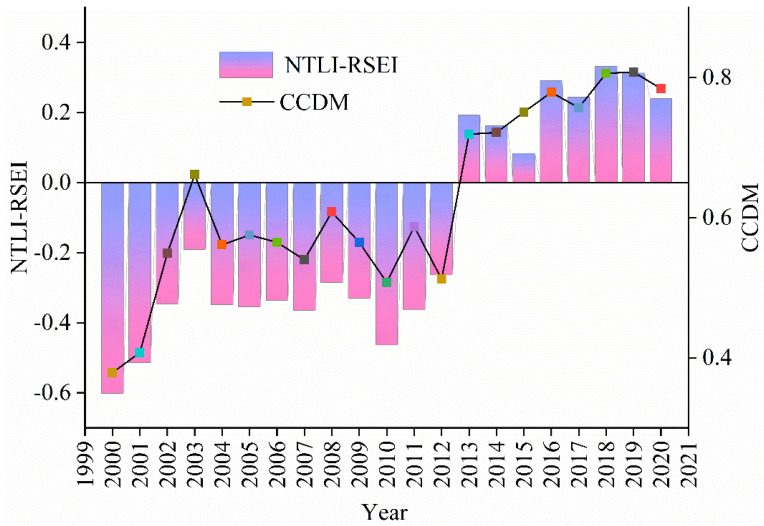
Finer division results between CCDM, NTLI, and RSEI.

Based on CCDM, the urban-ecological coupling type was further classified according to the relationship between NTLI and RSEI (Figure 8). Specifically, the normalized NTLI image subtracts RSEI, and the determination conditions are shown in Table 2. From 2000 to 2012, the NTLI is lower than the RSEI, indicating that the main urban area is lagging in urban development under a low degree of coupling and coordination, and the urban development is relatively backward. From 2013–2020, the NTLI is higher than the RSEI, indicating that the urban and ecological development is in a period of coupling and coordination, and the urban development is faster than the ecological improvement. CCDM has been at a high value in the past, but the city and the ecological environment were not in a ‘synchronous development state’. It suggested that urban ecology was not a simple indicator of change but a dynamic, continuous, and complex interactive process. The growth of urban areas has led to a reduction in green areas, an increase in population has led to a reduction in natural resources and an increase in ecological pressure.

### 4.4. Urbanization Development and the Continuing Dynamics of Ecological Space

**Figure 9:**
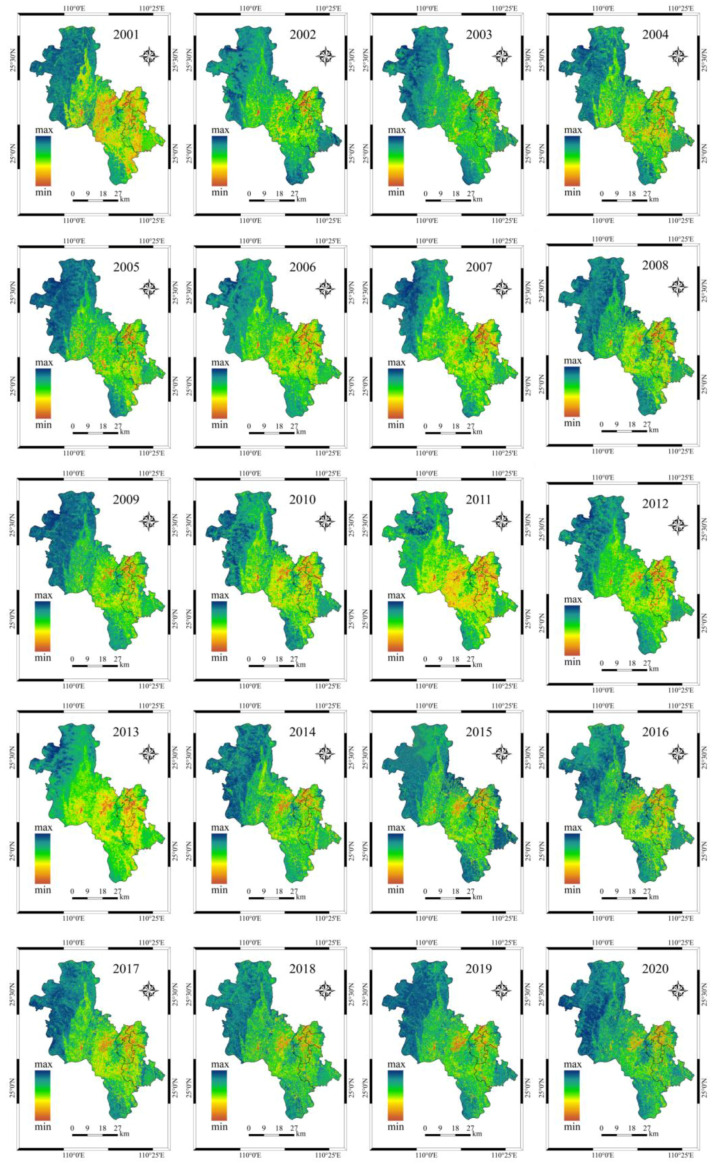
Annual spatial and temporal distribution of CCDM.

We used Formula (16) to calculate the spatial distribution of CCDM in the main urban area of Guilin in the past 20 years, as shown in Figure 9. Figure 9 illustrates the fine spatial distribution of urban development and ecology. CCDM showed apparent regional differences in spatial distribution between the urban center and the surrounding nature reserves. In the primary urban center, the level of urbanization was higher than the ecological level because of the large number of urban buildings, which showed a lagging ecological environment. Conversely, nature reserves in suburban areas with more vegetation and a lower level of urbanization presented a higher level of urban ecological coupling.

## 5. Discussions

When analyzing the urbanization level of the main urban area of Guilin, the paper integrated night light, population density, urbanization rate, and per capita GDP data, which could better reflect the comprehensive level of urbanization development than a single data index [45]. Because of Guilin’s special geographical location and urban positioning, its economic development is slow. For a long time, Guilin has been trying to reform its tourism industry, adhere to the national strategy of building Guilin into an international tourist destination, and strive to promote the transformation and upgrading of the tourism industry. Guilin’s leading role in tourism has been further enhanced.

In the past 20 years, Guilin has been committed to ecological construction. Relevant departments strengthened the management and protection of key ecological functional areas and natural reserves, implemented major ecological protection and restoration projects, and did a good job in the protection and restoration of important ecosystems and ecologically fragile areas. The protection of the Lijiang River has been strengthened. By 2020, the non-point source pollution problem in the urban section of the Lijiang River has been completely solved. We will improve the management system of the Lijiang River and establish a sound ecological compensation mechanism for the Lijiang River basin. This is also an important factor for the continuous improvement of the ecological environment in the main urban area of Guilin.

The coupling between city and ecological environment is a particularly complex problem. Past urban development in the main urban area of Guilin has had a certain impact on the ecological environment, which is also a problem that many cities will encounter in the process of development [46]. After the governance of Guilin Municipal Government, this contradiction has been alleviated, and even the improvement of the ecological environment has promoted the urbanization of Guilin. The two complement each other. It is undeniable that each indicator has its limitations. When discussing urbanization indicators, this paper only discusses population density, per capita GDP, urbanization rate, and night light density, but other studies show that land use, urban roads, etc. are becoming more and more important in urbanization construction [47,48].

## 6. Conclusions

(1) In order to ensure a better construction of the nighttime light index, this paper uses the 2013 monthly NPP/VIIRS and DMSP/OLS data to calibrate and simulate each other to obtain new nighttime light data. It was verified with the historical population (R^2^ = 0.72) and GDP data (R^2^ = 0.83). The total number of night lights in 2020 is 7.72 times higher than in 2000. NTLI grew to 0.90, and the urbanization of the urban area of Guilin was significant.
(2) RSEI was constructed to analyze 20 years of ecological changes in the urban area of Guilin through principal component analysis. RSEI increased from 0.45 in 2000 to 0.66 in 2020, with an overall increase in ecological quality. Spatial heterogeneity exists in some areas, mainly reflected in the ecological environment changes in the new and old urban areas. The rapid urban expansion and increased population activities in Lingui District have caused a continuous increase in ecological pressure and a decline in the quality of the ecological environment. On the contrary, some old urban areas, such as Qixing District, have improved ecological quality through urban transformation and greening construction. Among the four indicators, it is easy to find that the greenness and wetness indicators had a catalytic improvement effect on ecology, while dryness and heat had a negative destructive effect.
(3) The interactive relationship between urban development and the ecological environment improved from 2000 to 2020, when the stage of low coordination was transformed into a stage of highly coordinated development. A more delicate CCDM system can more accurately reflect the fine-grained urban-ecological interactions. It is worth noting that from 2013 onwards, the main urban area shifted from lagging urban development to lagging ecology. Since then, urban development has outpaced ecological development and constrained it.

### Policy Recommendations

In view of the above research conclusions, the following policy recommendations are proposed for reference:

(1) Strengthen the integrated development of urban agglomeration and narrow the internal development differences. From the perspective of urban development, the economy of Lingui District and Yanshan District is lower than that of other districts. Therefore, in terms of existing development elements, the Guilin government should appropriately increase the urban construction of these two districts.
(2) Adhere to the synchronous development of urbanization and ecological environment security. In the process of urban development in some regions, ecological damage has occurred. In the future, the Guilin government should strengthen the ecological protection of these regions. At the same time, we can rely on Guilin’s special tourism culture to develop the service industry to promote economic growth.

## Figures and Tables

**Table 1:** Corresponding wetness index coefficients for different sensors.

Sensor	Blue	Green	Red	Nir	Swir1	Swir2
TM	0.0315	0.2021	0.3102	0.1594	−0.6806	−0.6109
ETM	0.1509	0.1973	0.3102	0.1594	−0.6806	−0.6109
OLI	0.1511	0.1973	0.3283	0.3407	−0.7117	0.4559

**Table 2:** CCDM classification types.

CCDM Level	Subcategory Coordination	Systematic Exponential Comparison	Subcategory
0 ≤ CCDM ≤ 0.4	Low level of coordination	NTLI-RSEI > 0.1	Ecologically lagging
		|NTLI-RSEI| < 0.1	Urban development and ecological synchronization
		NTLI-RSEI < −0.1	Urban development lagging
0.4 ≤ CCDM ≤ 0.65	Moderate coordination	NTLI-RSEI > 0.1	Ecologically lagging
		|NTLI-RSEI| < 0.1	Urban development and ecological synchronization
		NTLI-RSEI < −0.1	Urban development lagging
0.65 ≤ CCDM ≤ 1.0	Highly coordinated	NTLI-RSEI > 0.1	Ecologically lagging
		|NTLI-RSEI| < 0.1	Urban development and ecological synchronization
		NTLI-RSEI < −0.1	Urban development lagging

**Table 3:** Night-time lighting data and historical yearbook statistics. Data sources: Statistics of Guangxi Zhuang Autonomous Region Statistics Bureau from 2000 to 2020 (http://tjj.gxzf.gov.cn/tjsj/tjnj (accessed on 10 September 2021)).

Year	IPD (People/km^2^)	GDP per Capita (¥)	Urbanization Rate(%)	Light Density at Night
2000	369	11,095.37	21.34	1.05
2001	382	12,282.15	21.80	1.11
2002	389	13,352.32	22.22	2.16
2003	397	14,516.69	22.57	3.16
2004	404	16,499.46	22.72	2.22
2005	411	19,035.93	22.95	2.30
2006	415	21,815.50	24.50	2.27
2007	421	26,026.88	23.63	2.08
2008	482	26,782.80	24.13	2.61
2009	484	28,704.59	23.94	2.27
2010	497	32,986.76	23.71	1.81
2011	503	37,831.78	23.58	2.36
2012	514	40,980.08	23.56	2.06
2013	522	45,453.81	23.45	4.48
2014	528	48,483.94	22.54	4.42
2015	537	51,051.46	30.51	4.47
2016	544	54,561.44	31.48	5.25
2017	551	51,640.27	33.37	4.94
2018	555	52,634.04	35.55	5.60
2019	558	57,415.71	48.13	5.57
2020	605	53,021.99	50.90	5.16

**Table 4:** Eigenvalues and contribution rates of the four indicators in PCA analysis.

Indicators	2000		2010		2020	
	Eigenvalue	Contribution Rate/(%)	Eigenvalue	Contribution Rate/(%)	Eigenvalue	Contribution Rate/(%)
PC1	0.3775	97.16	0.3659	95.15	0.3824	95.94
PC2	0.0048	98.40	0.0136	98.69	0.0125	99.08
PC3	0.0036	99.33	0.0040	99.73	0.0032	99.88
PC4	0.0026	100.0	0.0010	100.0	0.0005	100.0

**Table 5:** Normalized values for the four indicators and RSEI statistics.

Statistical Values	2000		2010		2020	
	Average	Standard	Average	Average	Average	Standard
NDVI	0.457	0.166	0.264	0.239	0.688	0.157
TCW	0.670	0.060	0.695	0.067	0.151	0.050
NDBSI	0.022	0.085	0.011	0.041	0.151	0.050
LST	0.403	0.055	0.856	0.124	0.274	0.079
RSEI	0.652	0.074	0.630	0.414	0.698	0.663

**Table 6:** RSEI class and area statistics.

Category	Class	2000–2004	2005–2010	2011–2015	2016–2020	2000–2020
Worse	−4 (km^2^)	2.0 (0.08%)	1.0 (0.04%)	2.7 (0.1%)	4.4 (0.2%)	1.1 (0.04%)
	−3 (km^2^)	12.4 (0.5%)	12.1 (0.5%)	17.7 (0.7%)	12.2 (0.5%)	16.2 (0.6%)
	−2 (km^2^)	61.2 (2.4%)	34.2 (1.4%)	55.4 (2.2%)	24.5 (1.0%)	51.6 (1.8%)
	−1 (km^2^)	396.5 (15.6%)	232.1 (9.2%)	212.9 (8.4%)	186.9 (7.4%)	129.6 (4.4%)
Unchanged	0 (km^2^)	1489.8 (59.1%)	976.6 (38.7%)	1582.1 (62.7%)	1015.1 (40.2%)	1105.3 (37.9%)
Better	1 (km^2^)	478.6 (18.9%)	1140.7 (45.2%)	554.5 (21.9%)	951.5 (37.7%)	691.7 (23.7%)
	2 (km^2^)	64.5 (2.6%)	109.3 (4.3%)	81.7 (3.2%)	271.4 (10.7%)	457.2 (15.7%)
	3 (km^2^)	16.1 (0.6%)	12.6 (0.5%)	13.4 (0.5)	43.3 (1.7%)	457.2 (15.7%)
	4 (km^2^)	1.4 (0.06%)	3.9 (0.2%)	2.1 (0.08%)	13.3 (0.5%)	11.0 (0.4%)

## Data Availability

Not applicable.

## Abbreviation

NTLI	night-time light index
RSEI	remote sensing ecological index
CCDM	coupling coordination degree model
NDVI	normalized difference vegetation index
PCA	principal component analysis
GEE	Google Earth Engine
TNL	total night-time light
TCW	tasseled cap wetness
NDBSI	normalized difference built-up and soil index
LST	land surface temperature
IBI	index-based built-up index
SI	soil index
C	coupling
IPD	initial population density
GDP	gross domestic product

## References

[1] Pickett S.T.A., Boone C.G., McGrath B.P., Cadenasso M.L., Childers D.L., Ogden L.A., McHale M., Grove J.M. (2013). Ecological science and transformation to the sustainable city. Cities.

[2] Zhang X., Wang Y., Qi Y., Wu J., Liao W., Shui W., Zhang Y., Deng S., Peng H., Yu X. (2016). Evaluating the trends of China’s ecological civilization construction using a novel indicator system. J. Clean. Prod..

[3] Feng R., Wang F., Wang K., Wang H., Li L. (2021). Urban ecological land and natural-anthropogenic environment interactively drive surface urban heat island: An urban agglomeration-level study in China. Environ. Int..

[4] He C., Gao B., Huang Q., Ma Q., Dou Y. (2017). Environmental degradation in the urban areas of China: Evidence from multi-source remote sensing data. Remote Sens. Environ..

[5] Hauser L.T., Féret J.-B., An Binh N., van der Windt N., Sil Â.F., Timmermans J., Soudzilovskaia N.A., van Bodegom P.M. (2021). Towards scalable estimation of plant functional diversity from Sentinel-2: In-situ validation in a heterogeneous (semi-)natural landscape. Remote Sens. Environ..

[6] Yan L., Zhang X., Pan H., Wu J., Lin L., Zhang Y., Xu C., Xu M., Luo H. (2021). Progress of Chinese ecological civilization construction and obstacles during 2003–2020: Implications from one set of emergy-based indicator system. Ecol. Indic..

[7] Chen M., Sui Y., Liu W., Liu H., Huang Y. (2019). Urbanization patterns and poverty reduction: A new perspective to explore the countries along the Belt and Road. Habitat Int..

[8] Cui X., Fang C., Liu H., Liu X. (2019). Assessing sustainability of urbanization by a coordinated development index for an Urbanization-Resources-Environment complex system: A case study of Jing-Jin-Ji region, China. Ecol. Indic..

[9] Zhou X., Yang L., Gu X., Zhang L., Li L. (2022). Scarcity Value Assessment of Ecosystem Services Based on Changes in Supply and Demand: A Case Study of the Yangtze River Delta City Cluster, China. Int. J. Environ. Res. Public Health.

[10] Destoumieux-Garzon D., Matthies-Wiesler F., Bierne N., Binot A., Boissier J., Devouge A., Garric J., Gruetzmacher K., Grunau C., Guegan J.F. (2022). Getting out of crises: Environmental, social-ecological and evolutionary research is needed to avoid future risks of pandemics. Environ. Int..

[11] Tang P., Huang J., Zhou H., Fang C., Zhan Y., Huang W. (2021). Local and telecoupling coordination degree model of urbanization and the eco-environment based on RS and GIS: A case study in the Wuhan urban agglomeration. Sustain. Cities Soc..

[12] Diksha, Kumar, A (2017). Analysing urban sprawl and land consumption patterns in major capital cities in the Himalayan region using geoinformatics. Appl. Geogr..

[13] Stokes E.C., Seto K.C. (2019). Characterizing urban infrastructural transitions for the Sustainable Development Goals using multi-temporal land, population, and nighttime light data. Remote Sens. Environ..

[14] Ali A., Alam Nayyar Z. (2021). A Modified Built-up Index (MBI) for automatic urban area extraction from Landsat 8 Imagery. Infrared Phys. Technol..

[15] Huang C., Wylie B., Yang L., Homer C., Zylstra G. (2010). Derivation of a tasselled cap transformation based on Landsat 7 at-satellite reflectance. Int. J. Remote Sens..

[16] Li X., Elvidge C., Zhou Y., Cao C., Warner T. (2017). Remote sensing of night-time light. Int. J. Remote Sens..

[17] Bennett M.M., Smith L.C. (2017). Advances in using multitemporal night-time lights satellite imagery to detect, estimate, and monitor socioeconomic dynamics. Remote Sens. Environ..

[18] Levin N., Kyba C.C.M., Zhang Q., Sánchez de Miguel A., Román M.O., Li X., Portnov B.A., Molthan A.L., Jechow A., Miller S.D. (2020). Remote sensing of night lights: A review and an outlook for the future. Remote Sens. Environ..

[19] Krikigianni E., Tsiakos C., Chalkias C. (2019). Estimating the relationship between touristic activities and night light emissions. Eur. J. Remote Sens..

[20] Asner G.P., Braswell B., Schimel D.S., Wessman C.A. (1998). Ecological Research Needs from Multiangle Remote Sensing Data. Remote Sens. Environ..

[21] Hao J., Xu G., Luo L., Zhang Z., Yang H., Li H. (2020). Quantifying the relative contribution of natural and human factors to vegetation coverage variation in coastal wetlands in China. Catena.

[22] Pettorelli N., Ryan S., Mueller T., Bunnefeld N., Jedrzejewska B., Lima M., Kausrud K. (2011). The Normalized Difference Vegetation Index (NDVI): Unforeseen successes in animal ecology. Clim. Res..

[23] Zhang C., Cheng Y., He H., Gao L., Liang J., Zhao X. (2017). Structural drivers of biomass dynamics in two temperate forests in China. Ecosphere.

[24] Brierley A.S., Kingsford M.J. (2009). Impacts of climate change on marine organisms and ecosystems. Curr. Biol..

[25] Niroumand-Jadidi M., Bovolo F., Bruzzone L. (2020). Water Quality Retrieval from PRISMA Hyperspectral Images: First Experience in a Turbid Lake and Comparison with Sentinel-2. Remote Sens..

[26] Painii-Montero V.F., Santillán-Muñoz O., Barcos-Arias M., Portalanza D., Durigon A., Garcés-Fiallos F.R. (2020). Towards indicators of sustainable development for soybeans productive units: A multicriteria perspective for the Ecuadorian coast. Ecol. Indic..

[27] Xu H., Wang M., Shi T., Guan H., Fang C., Lin Z. (2018). Prediction of ecological effects of potential population and impervious surface increases using a remote sensing based ecological index (RSEI). Ecol. Indic..

[28] Yue H., Liu Y., Li Y., Lu Y. (2019). Eco-Environmental Quality Assessment in China’s 35 Major Cities Based on Remote Sensing Ecological Index. IEEE Access.

[29] Cai J., Li X., Liu L., Chen Y., Wang X., Lu S. (2021). Coupling and coordinated development of new urbanization and agro-ecological environment in China. Sci. Total Environ..

[30] Xu D., Yang F., Yu L., Zhou Y., Li H., Ma J., Huang J., Wei J., Xu Y., Zhang C. (2021). Quantization of the coupling mechanism between eco-environmental quality and urbanization from multisource remote sensing data. J. Clean. Prod..

[31] Ariken M., Zhang F., Liu K., Fang C., Kung H.-T. (2020). Coupling coordination analysis of urbanization and eco-environment in Yanqi Basin based on multi-source remote sensing data. Ecol. Indic..

[32] Amani M., Ghorbanian A., Ahmadi S.A., Kakooei M., Moghimi A., Mirmazloumi S.M., Moghaddam S.H.A., Mahdavi S., Ghahremanloo M., Parsian S. (2020). Google Earth Engine Cloud Computing Platform for Remote Sensing Big Data Applications: A Comprehensive Review. IEEE J. Sel. Top. Appl. Earth Obs. Remote Sens..

[33] Li X., Li D., Xu H., Wu C. (2017). Intercalibration between DMSP/OLS and VIIRS night-time light images to evaluate city light dynamics of Syria’s major human settlement during Syrian Civil War. Int. J. Remote Sens..

[34] Goward S.N., Xue Y., Czajkowski K.P. (2002). Evaluating land surface moisture conditions from the remotely sensed temperature/vegetation index measurements. Remote Sens. Environ..

[35] Lobser S.E., Cohen W.B. (2007). MODIS tasselled cap: Land cover characteristics expressed through transformed MODIS data. Int. J. Remote Sens..

[36] Crist E.P. (1985). A TM Tasseled Cap equivalent transformation for reflectance factor data. Remote Sens. Environ..

[37] Huang H. (2020). Study on the Quality Development of New Urbanization with Chinese Characteristics.

[38] Baig M.H.A., Zhang L., Shuai T., Tong Q. (2014). Derivation of a tasselled cap transformation based on Landsat 8 at-satellite reflectance. Remote Sens. Lett..

[39] Xu H. (2008). A new index for delineating built-up land features in satellite imagery. Int. J. Remote Sens..

[40] Rikimaru A., Roy P.S., Miyatake S. (2002). Tropical forest cover density mapping. Trop. Ecol..

[41] Chander G., Markham B.L., Helder D.L. (2009). Summary of current radiometric calibration coefficients for Landsat MSS, TM, ETM+, and EO-1 ALI sensors. Remote Sens. Environ..

[42] Small C., Elvidge C.D., Balk D., Montgomery M. (2011). Spatial scaling of stable night lights. Remote Sens. Environ..

[43] Wang J., Liu H., Liu H., Huang H. (2021). Spatiotemporal Evolution of Multiscale Urbanization Level in the Beijing-Tianjin-Hebei Region Using the Integration of DMSP/OLS and NPP/VIIRS Night Light Datasets. Sustainability.

[44] Shen L., Huang Y., Huang Z., Lou Y., Ye G., Wong S.-W. (2018). Improved coupling analysis on the coordination between socio-economy and carbon emission. Ecol. Indic..

[45] Tian Y., Zhou D., Jiang G. (2020). Conflict or Coordination? Multiscale assessment of the spatio-temporal coupling relationship between urbanization and ecosystem services: The case of the Jingjinji Region, China. Ecol. Indic..

[46] Xie X., Sun H., Gao J., Chen F., Zhou C. (2021). Spatiotemporal Differentiation of Coupling and Coordination Relationship of Tourism–Urbanization–Ecological Environment System in China’s Major Tourist Cities. Sustainability.

[47] Gao L., Tao F., Liu R., Wang Z., Leng H., Zhou T. (2022). Multi-scenario simulation and ecological risk analysis of land use based on the PLUS model: A case study of Nanjing. Sustain. Cities Soc..

[48] Liu T.-Y., Su C.-W. (2021). Is transportation improving urbanization in China?. Socio-Econ. Plan. Sci..

